# Interspecies protein-protein interaction network construction for characterization of host-pathogen interactions: a *Candida albicans*-zebrafish interaction study

**DOI:** 10.1186/1752-0509-7-79

**Published:** 2013-08-16

**Authors:** Yu-Chao Wang, Che Lin, Ming-Ta Chuang, Wen-Ping Hsieh, Chung-Yu Lan, Yung-Jen Chuang, Bor-Sen Chen

**Affiliations:** 1 Laboratory of Control and Systems Biology, Department of Electrical Engineering, National Tsing Hua University, Hsinchu 30013, Taiwan; 2 Institute of Biomedical Informatics, National Yang-Ming University, Taipei 11221, Taiwan; 3 Institute of Communications Engineering, National Tsing Hua University, Hsinchu 30013, Taiwan; 4 Institute of Statistics, National Tsing Hua University, Hsinchu 30013, Taiwan; 5 Department of Life Science, National Tsing Hua University, Hsinchu 30013, Taiwan; 6 Institute of Molecular and Cellular Biology, National Tsing Hua University, Hsinchu 30013, Taiwan; 7 Department of Medical Science, National Tsing Hua University, Hsinchu 30013, Taiwan; 8 Institute of Bioinformatics and Structural Biology, National Tsing Hua University, Hsinchu 30013, Taiwan

**Keywords:** Computational systems biology, Network construction, Host-pathogen interaction, Protein-protein interaction network, Infection, Multivariate dynamic modeling, Redox

## Abstract

**Background:**

Despite clinical research and development in the last decades, infectious diseases remain a top global problem in public health today, being responsible for millions of morbidities and mortalities each year. Therefore, many studies have sought to investigate host-pathogen interactions from various viewpoints in attempts to understand pathogenic and defensive mechanisms, which could help control pathogenic infections. However, most of these efforts have focused predominately on the host or the pathogen individually rather than on a simultaneous analysis of both interaction partners.

**Results:**

In this study, with the help of simultaneously quantified time-course *Candida albicans*-zebrafish interaction transcriptomics and other omics data, a computational framework was developed to construct the interspecies protein-protein interaction (PPI) network for *C. albicans*-zebrafish interactions based on the inference of ortholog-based PPIs and the dynamic modeling of regulatory responses. The identified *C. albicans*-zebrafish interspecies PPI network highlights the association between *C. albicans* pathogenesis and the zebrafish redox process, indicating that redox status is critical in the battle between the host and pathogen.

**Conclusions:**

Advancing from the single-species network construction method, the interspecies network construction approach allows further characterization and elucidation of the host-pathogen interactions. With continued accumulation of interspecies transcriptomics data, the proposed method could be used to explore progressive network rewiring over time, which could benefit the development of network medicine for infectious diseases.

## Background

Despite clinical research and development in the last decades, infectious diseases remain a top global problem in public health today, being responsible for millions of morbidities and mortalities each year [1-3]. Investigating the infection process in detail can aid understanding of the mechanisms that underlie infection and the control of infection disease. To obtain an in-depth understanding of the infectious process, the specific interactions between the virulence factors of the invasive pathogen and the defensive mechanisms of the host need to be elucidated. *Candida albicans* is one of the most common fungal pathogens of medical importance [4]. In severe cases, *C. albicans* can penetrate through epithelial layers into deeper tissues and cause life-threatening systemic infections [5]. *C. albicans* can grow in a budded yeast form or in a highly polarized hyphal form; its yeast-to-hyphal transition ability in response to environmental changes is one of its most well-known virulence characteristics [6]. In addition to dimorphism, a number of fungal attributes, such as the expression of adhesion factors, directed growth/thigmotropism, stress adaptation, metabolic flexibility and the secretion of hydrolytic enzymes are also implicated in the infection process [7]. However, the exact molecular mechanisms by which *C. albicans* attaches to epithelial surfaces, invades various epithelial barriers, causes cell and tissue damage, and disseminates within the host are not fully understood [8].

Recently, zebrafish have been increasingly used in biomedical research due to their high reproductive rate, comprehensive molecular tools, and low maintenance costs [9,10]. Zebrafish are more similar to mammals than other mini-hosts (such as *Drosophila melanogaster*, *Galleria mellonella* and *Caenorhabditis elegans*) in terms of genetics, physiology, and anatomical structure, and most importantly, they have both innate and adaptive immune functions [11,12]. As a result, the zebrafish model has been used to study human pathogens or closely related animal pathogens, either using adult fish with a fully developed adaptive immune system, or using embryos or larvae that rely solely on innate immunity [13,14]. Chao *et al*. used zebrafish as a mini-vertebrate host system for their study of *C. albicans* infection, demonstrating that *C. albicans* can colonize and invade zebrafish at multiple anatomical sites and kill the fish in a dose-dependent manner [15]. Hence, zebrafish are suitable for our study characterizing host-pathogen interactions with *C. albicans*.

Host-pathogen interactions are enormously complex processes. While traditional biological research, which isolates and studies small sets of components, may provide some insights, these approaches are not well suited to address interaction mechanisms on a larger and more general scale [16]. To this end, a systems biology approach is an emerging strategy to better comprehend the underlying mechanisms that occur during host-pathogen interactions [17]. Indeed, several different systems biology approaches have demonstrated their effectiveness [18]. These approaches rely on an unbiased and global understanding of the transcriptomics of the host/pathogen. Further computational analyses of genome-wide gene expression profiles have partially revealed the mechanisms of interaction between host and pathogen, leading to a deeper understanding of the infection process [19]. Nevertheless, the majority of these studies have addressed the pathogen or host transcriptomics individually rather than simultaneously analyzing both interaction partners. Consequently, in this study, we aimed to analyze the host and pathogen simultaneously and consider the interacting host and pathogen as an orchestrated system.

During the dynamically changing environment of host-pathogen interactions, both host and pathogen have evolved numerous strategies for adaptation. These adaptations are mediated by complex interaction networks, which lead to changes to gene expression patterns. Consequently, we intended to elucidate the adaptation mechanisms by understanding the underlying interaction networks. Although several network construction schemes have been successfully applied to many biological scenarios, these have focused mainly on a single species. Recently, some computational prediction methods to infer host-pathogen interactions have been developed based on interologs [20,21] or gene expression profiles [22,23]. In this study, we developed a computational framework that integrated ortholog-based protein-protein interaction (PPI) inference and dynamic modeling of regulatory responses during *C. albicans* infections to construct the interspecies PPI network for characterization of host-pathogen interactions. With PPI data for two well-studied organisms, *S. cerevisiae* and *H. sapiens*, and the cross-species ortholog information among these species, we first inferred the candidate interspecies PPI network consisting of putative interspecies and intracellular PPIs. We then used multivariate dynamic models to describe the regulatory responses between pathogen and host in the infection process and to prune the candidate network based on the simultaneously quantified *C. albicans*-zebrafish interaction transcriptomics [24]. The identified *C. albicans*-zebrafish interspecies PPI network highlights the association between *C. albicans* pathogenesis and the zebrafish redox process, indicating that redox status is critical in the battle between the host and pathogen. With the accumulation of more interspecies transcriptomics data, the proposed interspecies network construction framework can be used to efficiently explore progressive network rewiring over time. Consequently, this proposed method could benefit the development of network medicine for infectious diseases.

## Methods

### Overview of the interspecies PPI network construction framework

**Figure 1:**
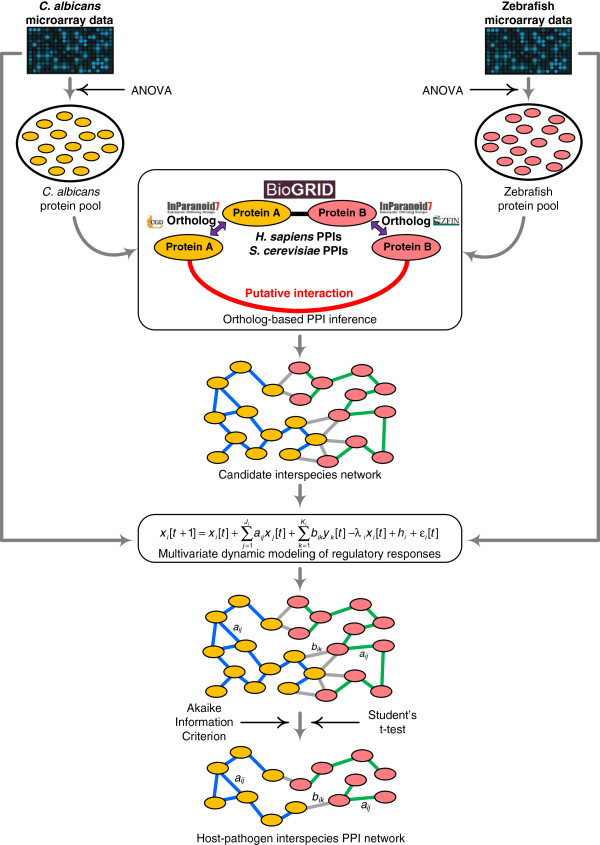
**Schematic overview of the interspecies protein-protein interaction network construction framework.** Protein-protein interaction (PPI) data from the BioGRID database, ortholog information from CGD, ZFIN, InParanoid, and simultaneous time-course microarray data for both *C. albicans* and zebrafish during *C. albicans*-zebrafish interactions were used for interspecies PPI network construction. On the basis of the PPI data for *S. cerevisiae* and *H. sapiens* and the ortholog information among these related species, putative interspecies and intracellular PPIs were inferred, which constitute the candidate interspecies network. Then, using multivariate dynamic modeling of regulatory responses and simultaneously quantified microarray data, the regulatory abilities were identified, and the significant interactions determined. In this manner, the candidate interspecies network was pruned to construct the refined host-pathogen interspecies PPI network. In the candidate interspecies network and the refined host-pathogen interspecies PPI network, yellow and pink nodes indicate *C. albicans* and zebrafish proteins, where blue, green, and grey edges denote *C. albicans* intracellular interactions, zebrafish intracellular interactions, and interspecies interactions, respectively.

The proposed interspecies PPI network construction framework is depicted in a schematic overview in Figure 1. The overall strategy is that we first infer the putative interspecies and intracellular PPIs among the proteins of interest and collect them as a candidate interspecies PPI network. Since the candidate interspecies network cannot accurately represent actual *C. albicans*-zebrafish interactions, it should be further validated and pruned. To this end, dynamic models are used to describe the regulatory responses of infection. With the help of simultaneous time-course microarray data for both *C. albicans* and zebrafish during *C. albicans*-zebrafish interactions, the regulatory abilities of interacting proteins in the multivariate dynamic models are identified. On the basis of these regulatory abilities, significant PPIs are determined and the candidate interspecies network is pruned, leading to the refined interspecies PPI network for *C. albicans*-zebrafish interactions. The details of the construction process are described in the following sections.

### Data mining and integration

This section describes the sources of all the data used in this study. Both zebrafish and *C. albicans* genome-wide microarray data were downloaded from the GEO database (GSE32119). Microarray experiments were performed to simultaneously profile genome-wide gene expressions in both *C. albicans* and zebrafish during the infection process. Adult AB strain zebrafish were intraperitoneally injected with 1 × 10^8^*C. albicans* (SC5314 strain) cells. Then, a two-step homogenization/mRNA extraction procedure was performing using the whole zebrafish infected with *C. albicans*. This approach could provide separate pools of gene transcripts from both the host and the pathogen, enabling individual estimation of specific gene expression profiles in either the host or the pathogen using sequence-targeted probes derived from the individual genome [24]. Agilent in situ oligonucleotide microarrays, which cover 6,202 and 26,206 genes for *C. albicans* and zebrafish respectively, were used to profile time-course gene expression at 9 time-points (0.5, 1, 2, 4, 6, 8, 12, 16, 18 hours post-infection) with three replicates for both organisms [24]. Manipulation of the animal model was approved by the Institutional Animal Care and Use Committee of National Tsing Hua University (IRB Approval No. 09808). In order to construct the interspecies PPI network for characterization of *C. albicans*-zebrafish interactions, the PPIs from *S. cerevisiae* and *H. sapiens* and the ortholog information among these species were used to infer the putative interspecies and intracellular PPI due to lack of sufficient information in the *C. albicans*, and zebrafish interactomes and their interspecies interactions. The PPI data for both *S. cerevisiae* and *H. sapiens* were obtained from the database of Biological General Repository for Interaction Datasets (BioGRID) (http://thebiogrid.org/) [25]. In BioGRID version 3.2.95, there are 89,445 non-redundant physical interactions among 15,690 proteins for *H. sapiens* and 75,065 non-redundant physical interactions among 6,043 proteins for *S. cerevisiae*. The ortholog information for the four species, namely, zebrafish, *H. sapiens*, *C. albicans* and *S. cerevisiae*, were acquired from the following databases: InParanoid (http://inparanoid.sbc.su.se/) [26], ZFIN (http://zfin.org/) [27], and the *Candida* Genome Database (CGD) (http://www.candidagenome.org/) [28]. The cellular information for both *C. albicans* and zebrafish proteins were retrieved from the Gene Ontology (GO) (http://www.geneontology.org/) [29], CGD, and ZFIN databases.

### Selection of protein pool

The first step of interspecies PPI network construction is to select proteins of interest for both host and pathogen. Generally, selection of proteins can be divided into two categories: expression-based selection and function-based selection. For expression-based selection, statistical methods such as one-way analysis of variance (ANOVA) or simply fold change selection are usually applied to gene expression profiles from microarray experiments or RNA sequencing (RNA-seq) for global selection of genes/proteins of interest. In this case, the constructed network will represent the global scenario for all the dynamically regulated genes/proteins under the experimental condition. On the other hand, the function-based selection method is applied only if we want to construct the network for specific functions. GO annotations are useful tools for functional annotation of genes/proteins. In this study, one-way ANOVA was employed to detect significant gene expression variations across nine time-points for each gene. In this manner, the dynamically regulated genes in both *C. albicans* and zebrafish could be selected for global characterization of *C. albicans*-zebrafish interactions. The null hypothesis of ANOVA assumed that the average expression level of a gene would be the same at every time point [30]. Genes with Bonferroni-adjusted *p*-values of less than 0.05 were identified as dynamically regulated genes and their corresponding gene products were selected in the protein pool as target proteins.

### Inference of putative interspecies and intracellular PPIs

After the selection of proteins of interest for both *C. albicans* and zebrafish, we then sought to identify the candidate interspecies PPI network among these selected proteins. However, due to extremely low coverage of the *C. albicans* and zebrafish interactomes and lack of interspecies PPIs between *C. albicans* and zebrafish, ortholog-based PPI prediction was used to infer the putative PPIs within and between *C. albicans* and zebrafish [20,21,31]. The PPI data of *S. cerevisiae* and *H. sapiens* from BioGRID and the ortholog information among these species from the InParanoid, CGD, and ZFIN databases were used to infer the putative interspecies and intracellular PPIs. The concept of the ortholog-based PPI inference is shown in Figure 1. For example, suppose that protein A’ and protein B’ of *S. cerevisiae* (or *H. sapiens*) are shown to interact based on the BioGRID database. From the InParanoid and CGD databases, we further identify that *C. albicans* protein A is orthologous to protein A’; from the InParanoid and ZFIN databases, we identify that zebrafish protein B is orthologous to protein B’. Based on the data mining via these databases, we infer that protein A in *C. albicans* and protein B in zebrafish are a putative interspecies PPI pair. Similarly, the putative intracellular PPIs can also be predicted for both *C. albicans* and zebrafish. Following this data mining methodology, the putative interspecies and intracellular PPIs were inferred and the candidate interspecies PPI network can be constructed by simply linking proteins inferred by our proposed method to interact with each other (Figure 1). It should be noted that the putative interspecies and intracellular PPIs inferred from the ortholog-based method were derived under many different experimental conditions, which cannot accurately reflect the actual condition of host-pathogen interactions during *C. albicans* infections; that is, false positives may be present among these putative PPIs. Therefore, these putative PPIs should be further validated by time-series microarray data of *C. albicans*-zebrafish interactions as described in the following section.

### Multivariate dynamic modeling of regulatory responses during *C. albicans* infections

In order to validate the putative PPIs and to prune the candidate interspecies network obtained above using the simultaneously quantified *C. albicans*-zebrafish interaction transcriptomics, dynamic models were employed to describe the regulatory responses of infection. For both *C. albicans* and zebrafish, the gene expression of target protein *i* in the candidate interspecies network can be described by the following multivariate linear dynamic model:

(1) $$\begin{array}{ll} x_{i}\left[t+1\right]=x_{i}\left[t\right] & +\sum_{j=1}^{J_{i}}a_{\mathrm{ij}}x_{j}\left[t\right]+\sum_{k=1}^{K_{i}}b_{\mathrm{ik}}y_{k}\left[t\right]-\lambda_{i}x_{i}\left[t\right]\\ & +h_{i}+\epsilon_{i}\left[t\right] \end{array}$$

where *x*_*i*_[*t*] represents the mRNA expression level at time *t* for gene *i* of the corresponding target protein *i* (*i* = 1, 2, …, *N*), *a*_*ij*_ denotes the regulatory ability of the *j*-th intracellular interactive protein to the *i***-**th target protein, *x*_*j*_[*t*] represents the mRNA expression level of the *j***-**th intracellular protein interacting with the target protein *i*, *b*_*ik*_ denotes the regulatory ability of the *k***-**th interspecies interactive protein to the *i***-**th target protein, *y*_*k*_[*t*] represents the mRNA expression level of the *k***-**th interspecies protein interacting with the target protein *i*, *λ*_*i*_ indicates the degradation effect of the target protein *i*, *h*_*i*_ represents the basal expression level, *ϵ*_*i*_[*t*] represents the stochastic noise, and *J*_*i*_ and *K*_*i*_ denote the numbers of intracellular and interspecies proteins interacting with target protein *i* in the candidate interspecies PPI network. In other words, only the proteins interacting with target protein *i* in the candidate interspecies PPI network were described in the multivariate linear dynamic model, therefore constraining the multivariate dynamic model based on the candidate interspecies network. In addition, it should be noted that only the mRNA expression level of the corresponding target protein were used in this equation, not the concentrations of the proteins. The biological interpretation of equation (1) is that the mRNA expression level for gene *i* of the corresponding target protein *i* at the next time *t* + 1 is determined by the current gene expression level, the regulatory effects of *J*_*i*_ intracellular interactive proteins, the regulatory effects of *K*_*i*_ interspecies interactive proteins, the degradation of the present state, the basal protein level from other sources beyond the interactive proteins in the system, and some stochastic noises. For each target protein with putative PPIs in the candidate interspecies network, a dynamic model was constructed. Consequently, a set of dynamic equations for all the target proteins can be used to describe the entire candidate interspecies PPI network.

### Identification of regulatory abilities and determination of significant interactions

From the network point-of-view, the interspecies PPI network depicted by the multivariate linear dynamic models in equation (1) represents how *C. albicans* and zebrafish interact during the infection process. Once *C. albicans* invades zebrafish tissues and initiates the infection process as the interaction between pathogen and host, some interacting proteins between *C. albicans* and zebrafish become active. These interspecies interactions are captured by the term $\sum_{k=1}^{K_{i}}b_{\mathrm{ik}}y_{k}\left[t\right]$ in equation (1); the response of intracellular protein interactions is instead reflected through the term $\sum_{j=1}^{J_{i}}a_{\mathrm{ij}}x_{j}\left[t\right]$. In other words, the regulatory abilities, specifically, the *b*_*ik*_ and *a*_*ij*_ terms, indicate the weighting of the edges in the interspecies PPI network. Hence, it is essential to identify these regulatory abilities and determine the significant interactions during *C. albicans* infections such that the candidate interspecies network can be further pruned into the refined interspecies PPI network that accurately captures *C. albicans*-zebrafish interactions during the infection process.

With the help of simultaneously quantified time-course microarray data for both *C. albicans* and zebrafish during the infection process, identification of parameter in the candidate interspecies network was performed protein by protein. Since the basal expression level *h*_*i*_ in equation (1) should always be non-negative, some constraints should be employed when identifying the system parameters. Therefore, the system parameters were identified by solving the constrained least squares problem [32,33]. The multivariate linear dynamic model in equation (1) can be rewritten as the following regression form:

(2) $$\begin{array}{cc} x_{i}\left[t+1\right] & =\left[\begin{array}{llllllll} x_{1}\left[t\right] & \cdots & x_{J_{i}}\left[t\right] & y_{1}\left[t\right] & \cdots & y_{K_{i}}\left[t\right] & x_{i}\left[t\right] & 1 \end{array}\right]\cdot \left[\begin{array}{c} a_{i1}\\ \vdots \\ a_{iJ_{i}}\\ b_{i1}\\ \vdots \\ b_{iK_{i}}\\ \left(1-\lambda_{i}\right)\\ h_{i} \end{array}\right]+\epsilon_{i}[t]\\ & \equiv \phi_{i}^{T}\left[t\right]\cdot \theta_{i}+\epsilon_{i}[t] \end{array}$$

where *ϕ*_*i*_[*t*] denotes the regression vector which can be obtained from the data and *θ*_*i*_ is the parameter vector to be estimated for target protein *i*. In order to avoid overfitting the estimated parameters, the original data points (9 time-points from the original microarray data) were interpolated to *L* data points by the cubic spline method (*L* roughly equals to 5 times the number of parameters that need to be identified, namely, 5(*J*_*i*_ + *K*_*i*_ + 2) for target protein *i* since the parameters to be identified are $a_{i1}\cdots a_{iJ_{i}},b_{i1}\cdots b_{iK_{i}},\lambda_{i},\;\mathrm{and}\;h_{i}$). In other words, there were {*x*_*i*_[*l* + 1], *ϕ*_*i*_[*l*]} data point pairs for *l* ∈ {1, …, *L* − 1}. Hence, equation (2) can be written in the following form for target protein *i*:

(3) $$X_{i}=\Phi_{i}\cdot \theta_{i}+{Ε}_{i}$$

where

$$X_{i}=\left[\begin{array}{c} x_{i}\left[2\right]\\ \vdots \\ x_{i}\left[L\right] \end{array}\right],\;\Phi_{i}=\left[\begin{array}{c} \phi_{i}^{T}\left[1\right]\\ \vdots \\ \phi_{i}^{T}\left[L-1\right] \end{array}\right],\;{Ε}_{i}=\left[\begin{array}{c} \epsilon_{i}\left[1\right]\\ \vdots \\ \epsilon_{i}\left[L-1\right] \end{array}\right]$$

In this manner, the parameter estimation problem for target protein *i* in the candidate interspecies network (equation (3)) can be represented by the following constrained least-squares minimization equation [33]:

(4) $$\min_{\theta_{i}}\frac{1}{2}{ǁ\Phi_{i}\cdot \theta_{i}-X_{i}ǁ}_{2}^{2}\;\mathrm{such}\;\mathrm{that}\;A\cdot \theta_{i}\leq b$$

where $A=\mathrm{diag}\left[\begin{array}{ccccc} 0 & \cdots & 0 & 0 & -1 \end{array}\right]$ and $b={\left[\begin{array}{ccc} 0 & \cdots & 0 \end{array}\right]}^{T}$, constraining the parameters *h*_*i*_ to be non-negative.

Once the system parameters for all proteins in the candidate interspecies network were identified using equation (4), the significant protein interactions could be determined based on the estimated regulatory abilities (the *b*_*ik*_ and *a*_*ij*_ terms). Akaike Information Criterion (AIC) [32,34] and the Student’s t-test [30] were applied for system order selection and for determining significance of the protein interactions. AIC, which includes both estimated residual error and model complexity in one statistic, quantifies the relative goodness of fit of a model. For a protein interaction model with *J*_*i*_ + *K*_*i*_ interaction parameters (or proteins) to fit with data from *L* samples, the AIC can be written as follows [32,34]:

(5) $$\begin{array}{ll} \mathrm{AIC}\left(J_{i}+K_{i}\right)= & \log\left(\frac{1}{L}{\left(X_{i}-{\hat{X}}_{i}\right)}^{T}\left(X_{i}-{\hat{X}}_{i}\right)\right)\\ & +\frac{2\left(J_{i}+K_{i}\right)}{L} \end{array}$$

where ${\hat{X}}_{i}$ denotes the estimated expression profile of the *i*-th target protein, that is, ${\hat{X}}_{i}=\Phi_{i}\cdot {\hat{\theta }}_{i}$, and ${\hat{\sigma }}_{i}{}^{2}=\frac{1}{L}{\left(X_{i}-{\hat{X}}_{i}\right)}^{T}\left(X_{i}-{\hat{X}}_{i}\right)$ is the estimated residual error. As the residual error ${\hat{\sigma }}_{i}^{2}$ decreases, the AIC decreases. In contrast, while the number of interactive proteins (or parameters) *J*_*i*_ + *K*_*i*_ increases, the AIC increases. Therefore, there is a tradeoff between residual error and model order. As the expected residual error decreases with increasing number of interactive proteins in models of inadequate complexity, there should be a minimum around the optimal interactive protein number. The minimization achieved in equation (5) will indicate the ideal model order (namely, the optimal number of proteins that interact with the target protein) of the protein interaction system. With the statistical selection of *J*_*i*_ + *K*_*i*_ interactive proteins by minimization of the AIC, the question of whether an interactive protein is a significant one or just a false positive for the *i*-th target protein can be determined. Hence, AIC can be adopted to select system order, filtering out insignificant protein interactions in the candidate interspecies network based on the estimated regulatory abilities (*b*_*ik*_ and *a*_*ij*_ terms). Once the estimated regulatory abilities were examined using the AIC model selection criteria, the Student’s t-test was further applied to determine the statistical significance of the parameters. The *p*-values for the regulatory abilities were calculated under the null hypothesis *H*_0_ : *b*_*ik*_ = 0 or *H*_0_ : *a*_*ij*_ = 0 [30]. The interactions with Bonferroni-adjusted *p*-value≦0.05 were identified as significant interactions and preserved in the refined interspecies PPI network. In this manner, insignificant interactions in the candidate interspecies network were pruned to construct the refined host-pathogen interspecies PPI network.

## Results

### Construction of interspecies PPI network

**Figure 2:**
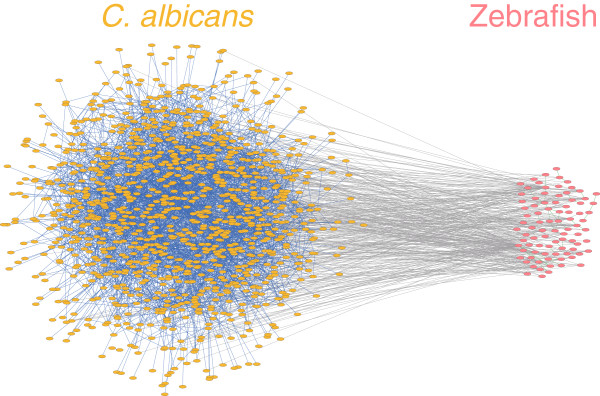
**The constructed *C. albicans*-zebrafish interspecies protein-protein interaction network.** There were 371 interspecies interactions, 3,504 *C. albicans* intracellular interactions, and 35 zebrafish intracellular interactions among 1,127 *C. albicans* proteins and 87 zebrafish proteins in the constructed interspecies network [see Additional file 1]. Representation of color nodes and edges are the same as in Figure 1. The figure was created by Cytoscape [35] and the protein names were omitted for simplicity.

In this study, our main objective was to identify key host**-**pathogen interactions during the infection process for better understanding of adaptation mechanisms during the battle between host and pathogen. As shown in Figure 1, various kinds of omics data and databases were mined and integrated as the input for the proposed interspecies PPI network construction method, including microarray gene expression data, ortholog information, and PPI data. On the basis of the time-course gene expression profiles and one-way ANOVA, 1,728 genes (27.86%) for *C. albicans* and 680 genes (2.59%) for zebrafish were identified as dynamically regulated genes and their corresponding gene products were selected as target proteins in the protein pool. Then, the putative interspecies and intracellular interactions among these target proteins were inferred and a candidate interspecies network was built. There were 1,606 putative interspecies interactions, 17,456 putative intracellular interactions for *C. albicans*, and 75 putative intracellular interactions for zebrafish among 1,230 *C. albicans* proteins and 130 zebrafish proteins in the candidate interspecies network. It should be noted that the target proteins without inferred protein interactions were excluded from the candidate interspecies network and further analysis. Next, a multivariate linear dynamic model was used as a mathematical description for the regulatory responses of the candidate interspecies network. On the basis of this multivariate linear dynamic model and the simultaneously quantified time-course transcriptomics, a refined *C. albicans*-zebrafish interspecies PPI network was constructed during the infection process. In the constructed interspecies network, there were 371 interspecies interactions, 3,504 intracellular interactions for *C. albicans*, and 35 intracellular interactions for zebrafish among 1,127 *C. albicans* proteins and 87 zebrafish proteins (Figure 2 and Additional file 1). Since the focus of this study lies in the interaction mechanisms between the host and the pathogen, the identified novel interspecies host-pathogen interactions, rather than the intracellular interactions, were further investigated in the following section.

### Novel interspecies interactions highlight the association between *C. albicans* pathogenesis and the zebrafish redox process

**Figure 3:**
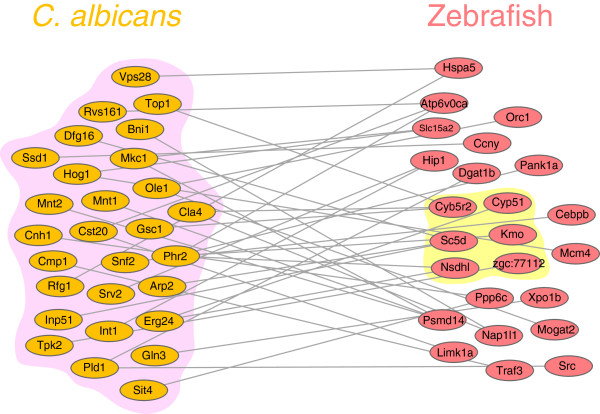
**The interspecies subnetwork for *C. albicans *pathogenesis proteins.** This figure indicates the *C. albicans* pathogenesis subnetwork extracted from the interspecies PPI network in Figure 2. The 24 zebrafish proteins interacting with *C. albicans* pathogenesis proteins (purple shadow) were found to be statistically enriched with proteins annotated with oxidation-reduction process (yellow shadow) (*p* < 0.01), highlighting the association between *C. albicans* pathogenesis and the zebrafish redox process. The intracellular protein interactions for both *C. albicans* and zebrafish were omitted for simplicity.

On the basis of the proposed computational framework, several novel interspecies interactions were identified in the constructed host-pathogen interspecies PPI network. Since the interspecies network construction method was proposed to elucidate pathogenic and defensive mechanisms during the infection process, the interspecies subnetwork for *C. albicans* virulence proteins, namely, proteins annotated with GO term pathogenesis, were further investigated. From these identified interspecies PPIs, 24 zebrafish proteins were found to interact with *C. albicans* virulence proteins (Figure 3). In addition, oxidation-reduction process was the only significant GO term shared among these 24 proteins (*p <* 0.01, Fisher’s exact test), highlighting the association between *C. albicans* pathogenesis and the zebrafish redox process. Six zebrafish proteins in the pathogenesis subnetwork, that is, Cyb5r2, Cyp51, Kmo, Nsdhl, Sc5d, and zgc:77112, were annotated with oxidation-reduction process (Figure 3) and all of their gene expressions were repressed over time, except for zgc:77112 [see Additional file 2]. Host defense against *C. albicans* infection relies mainly on phagocytes of the innate immune system, and one important host response generated by phagocytes is the production of reactive oxygen species (ROS). Free oxygen radicals produced by the oxidation-reduction process are highly toxic to pathogens and are utilized for pathogen clearance. In addition, ROS has been demonstrated to act as secondary signaling molecules contributing to signaling cascades related to inflammation, apoptosis, and immune responses [36]. For example, in certain cell lines, activation of the proinflammatory transcription factor NF-κB is dependent on ROS [37]. Therefore, immune cells depend on ROS to not only kill phagocytosed pathogens directly, but also to mediate inflammatory and immune signaling pathways [36]. As a result, a wide variety of pathogens have developed various molecular strategies to prevent host ROS generation, including *Helicobacter pylori*, *Legionella pneumophila*, and *Aspergillus fumigatus*[38-40]. Through these ROS inhibition mechanisms, these pathogenic organisms evade host immune responses. Similarly, *C. albicans* has the ability to suppress ROS production in host immune cells [41]. The precise mechanism utilized by *C. albicans* remains unclear; however, *C. albicans* catalase and surface superoxide dismutase have been implicated in counteracting the oxidative burst from phagocytes [42,43]. Since ROS also carry out important signaling functions as stated above, suppression of ROS production by *C. albicans* may not only result in evasion of phagocytic killing, but also in significant modulation of anti-*Candida* inflammatory responses, which directly benefits the pathogen further. As a result, suppression of ROS production may represent an important immune evasion mechanism for *C. albicans*.

On the other hand, infection by some pathogens such as *Entamoeba histolytica* and Japanese encephalitis virus results in enhanced ROS formation [44,45]. These pathogens have been reported to utilize enhanced ROS generation to induce host cell death, thus allowing themselves to escape the cell. This mechanism is likely to contribute to the spread of the pathogens [36]. Recently, it has been demonstrated that *Sclerotinia sclerotiorum*, a fungal pathogen that infects virtually all dicotyledonous plants, can both suppress and induce host ROS formation during infection via the secretion of oxalic acid [46]. During the initial stages of infection, *S. sclerotiorum* dampens the oxidative burst of the plant and generates a reducing environment in host cells. Once infection is established, the pathogen induces the generation of plant ROS (oxidizing conditions) leading to programmed cell death of the host, which directly benefits the pathogen [46]. In this manner, *Sclerotinia* uses a novel strategy involving regulation of host redox status to establish infection. Although there is no evidence that *C. albicans* is capable of inducing ROS production in host cells to date (Wellington *et al.* demonstrated that *C. albicans* suppresses production of ROS in phagocytes within 180 minutes of infection [41]), we postulated that *C. albicans* may also modulate ROS levels to subvert immune defenses in the same way as *S. sclerotiorum*, that is, by suppressing host ROS production in the initial stages of infection and inducing ROS in the later stages. Further experiments measuring ROS at different infection stages are needed to validate this hypothesis. Taken together, novel interspecies interactions identified in this study highlight the association between *C. albicans* pathogenesis and the zebrafish redox process. The redox status in both the host and pathogen can be a critical factor that determines the outcome of the battle between the host and pathogen. From the perspective of the pathogen, it is essential to be in control of the redox environment and the cell death pathways of the host in order to subvert immune defenses by the host and support self-survival. In contrast, during an immune response, the host seeks to control the redox environment and the cell death pathways to the detriment of the pathogen.

## Discussion

Infectious disease is one of the leading causes of death worldwide, and complex interaction mechanisms between host and pathogen underlie the process of infection. However, most studies exploring host-pathogen interactions have predominately focused on the host or the pathogen individually rather than simultaneously analyzing both interaction partners. Although these single-species studies have provided insights into the pathogenic and defensive mechanisms for host-pathogen interactions, they did not give clues about interspecies functional associations between host and pathogen. Detailed knowledge of host-pathogen protein interactions may enable us to comprehend the mechanisms of infection and to identify better strategies to prevent or cure infection [47]. Accordingly, in this study, we developed a computational framework to efficiently construct the interspecies PPI network focusing on the characterization of interspecies interactions between host and pathogen. Based on ortholog-based PPI inference and multivariate dynamic modeling of regulatory responses during *C. albicans* infections, several omics data were integrated for interspecies PPI network construction. The proposed computational method has been shown to be useful, emphasizing the association between *C. albicans* pathogenesis and the zebrafish redox process, and the idea that redox status is critical during the battle between the host and pathogen. According to our findings and evidence from other species, we also speculated that *C. albicans* may suppress host ROS production in the initial stages of infection and induce ROS formation in the later stages to subvert the host immune defense. However, further experiments are required to validate this hypothesis. Previous studies have demonstrated that hyphal morphogenesis is an important virulence factor in *C. albicans*[6,48]. Therefore, in addition to the pathogenesis subnetwork, we also explored zebrafish proteins that interacted with *C. albicans* hyphae-related proteins, specifically, proteins annotated with GO term hyphal growth, in the constructed interspecies network. However, only the general GO terms, such as metabolic process and lipid biosynthetic process, were significantly enriched among those zebrafish proteins. Because these general biological processes add little to the understanding of the adaptation mechanisms during the battle between host and pathogen, they are not discussed in the current study. Although the proposed interspecies network construction method was shown to be useful, some improvements remain to be addressed. Due to extremely low coverage of the *C. albicans* and zebrafish interactomes and lack of interspecies PPIs between *C. albicans* and zebrafish, ortholog-based PPI prediction was used to infer the putative PPIs among and between *C. albicans* and zebrafish. Although using interologs to infer host-pathogen interaction has been shown to be a useful approach [20], the putative PPIs may still contain inaccuracies, which could lead to deviation of the constructed interspecies network from the actual network. Therefore, high coverage of the *C. albicans* and zebrafish interactomes or even the experimentally validated interspecies PPIs will improve the network construction scheme.

With the interspecies PPI network construction scheme, we are able to construct interspecies networks for all kinds of interacting organisms efficiently given the interspecies transcriptomics data. In addition, the constructed interspecies network can be easily scalable, that is, the use of the computational scheme is not limited by the number of proteins of interest. Recently, Tierney *et al.* used simultaneous RNA-seq to quantify *C. albicans* and *Mus musculus* gene expression dynamics during phagocytosis by dendritic cells and inferred an interspecies regulatory network that also identified novel interspecies host-pathogen interactions [49]. On the basis of their inferred network, they proposed a mechanism whereby murine Ptx3 binding to *C. albicans* leads to cell wall remodeling via fungal Hap3 target genes, thereby altering recognition of the fungus by immune cells and attenuating host immune responses [49]. Their work successfully demonstrated the usefulness of network inference approaches to decipher microbial pathogenesis mechanisms. Nevertheless, their network inference method was restricted to a limited number of genes with prior knowledge, which can be overcome by our proposed computational scheme. Advancing from the investigation of single species, the interspecies network construction approach can further help characterize and elucidate host-pathogen interactions. With the accumulation of interspecies transcriptomics data, the proposed framework can be used to explore progressive network rewiring over time. In this manner, the dynamics of the interspecies system can be comprehensively studied. It has been suggested that a disease is rarely a consequence of an abnormality in a single gene/protein given the functional interdependencies between molecular components in the cell [50], and that both network connectivity and dynamics are important targets for therapeutic intervention [51]. Consequently, we believe that network medicine targeting network dynamics can be developed for infectious diseases with the help of the proposed interspecies protein interaction construction method.

## Conclusions

In this study, a computational framework which integrated multiple omics data was proposed to construct an interspecies PPI network for characterization of host-pathogen interactions. The proposed method was shown to be useful, with results highlighting an association between *C. albicans* pathogenesis and the zebrafish redox process during *C. albicans* infection in zebrafish. Results further indicated that redox status is critical during the battle between the host and pathogen, which could determine the outcome of infection. While the pathogen can control the redox environment to subvert immune defenses and support self-survival, in contrast, the host controls the redox environment to the detriment of the pathogen during an immune response. With continued accumulation of interspecies transcriptomics data, the proposed method could be helpful in the development of network medicine for infectious diseases from an interspecies network perspective.

## Competing interests

The authors declare that they have no competing interests.

## Authors’ contributions

YCW developed the method, performed the analysis, evaluated the results, and wrote the manuscript. CL helped to develop the method and revised the manuscript. MTC participated in the method development and helped to draft the manuscript. WPH participated in the design of the study and performed the statistical analysis. CYL and YJC conceived of the study, participated in its design, and provided essential guidance. BSC conceived of the study, provided essential guidance and revised the manuscript. All authors read and approved the final manuscript.

## Supplementary Material

Additional file 1 **The constructed *C. albicans*-zebrafish interspecies network.** The intracellular and interspecies PPIs among and between *C. albicans* and zebrafish in the constructed interspecies network are listed in the file. Click here for file

Additional file 2 Gene expression profiles of zebrafish proteins annotated with oxidation-reduction process in the pathogenesis subnetwork. Click here for file
