# Supplementary material for: Interspecies protein-protein interaction network construction for characterization of host-pathogen interactions: a Candida albicans-zebrafish interaction study
Source: BMC Syst Biol. 2013 Aug 16;7:79. doi: 10.1186/1752-0509-7-79 (PMC3751520; doi:10.1186/1752-0509-7-79)

Additional file 2 for

Interspecies protein-protein interaction network construction for characterization of host-pathogen interactions: a *Candida albicans*-zebrafish interaction study

Gene expression profiles of zebrafish proteins annotated with oxidation-reduction process in the pathogenesis subnetwork

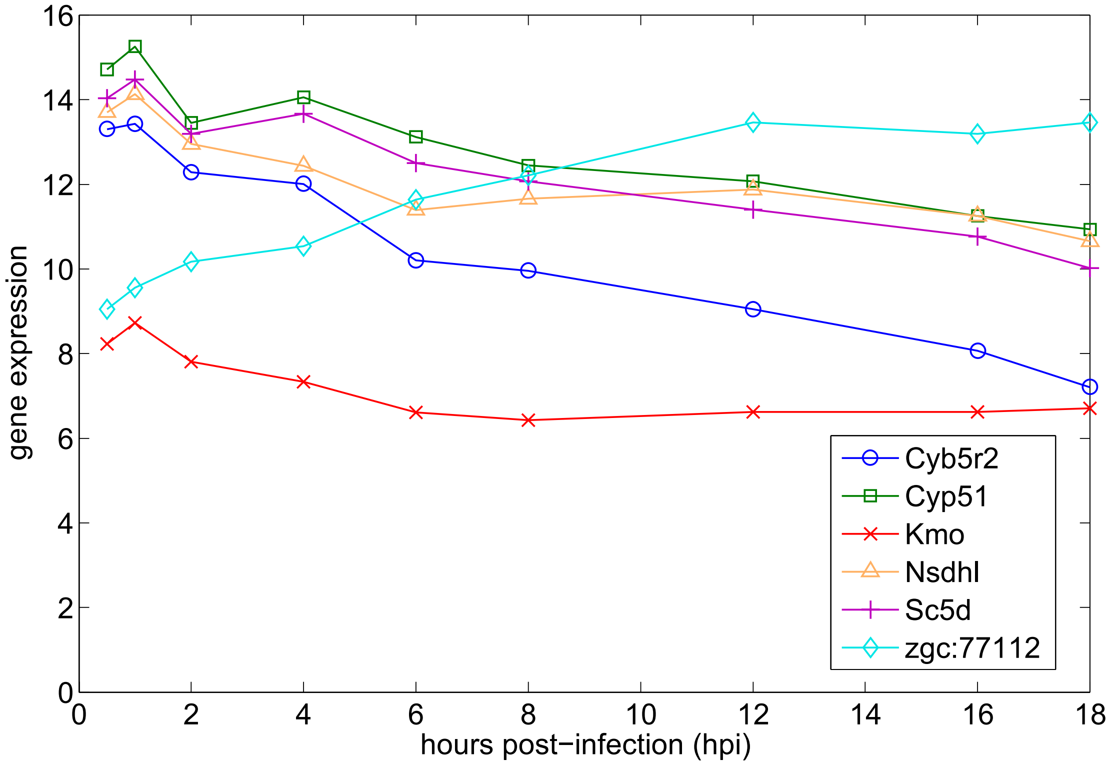

Supplement: Additional file 2 — Gene expression profiles of zebrafish proteins annotated with oxidation-reduction process in the pathogenesis subnetwork. [file 1752-0509-7-79-S2.pdf]
